# Cancer cachexia and its pathophysiology: links with sarcopenia, anorexia and asthenia

**DOI:** 10.1002/jcsm.12528

**Published:** 2020-03-06

**Authors:** Sara Peixoto da Silva, Joana M.O. Santos, Maria Paula Costa e Silva, Rui M. Gil da Costa, Rui Medeiros

**Affiliations:** ^1^ Molecular Oncology and Viral Pathology Group, IPO Porto Research Center (CI‐IPOP) Portuguese Oncology Institute of Porto (IPO Porto) Porto Portugal; ^2^ Faculty of Medicine of the University of Porto (FMUP) Porto Portugal; ^3^ Palliative Care Service Portuguese Oncology Institute of Porto (IPO Porto) Porto Portugal; ^4^ Center for the Research and Technology of Agro‐Environmental and Biological Sciences (CITAB) University of Trás‐os‐Montes and Alto Douro (UTAD) Vila Real Portugal; ^5^ Postgraduate Programme in Adult Health (PPGSAD) and Tumour Biobank Federal University of Maranhão (UFMA) São Luís Brazil; ^6^ Virology Service Portuguese Oncology Institute of Porto (IPO Porto) Porto Portugal; ^7^ Biomedical Research Center (CEBIMED) Faculty of Health Sciences of the Fernando Pessoa University Porto Portugal; ^8^ Research Department Portuguese League Against Cancer ‐ Regional Nucleus of the North (Liga Portuguesa Contra o Cancro – Núcleo Regional do Norte) Porto Portugal

**Keywords:** Cachexia, Sarcopenia, Anorexia, Asthenia, Muscle wasting, Cancer

## Abstract

Cancer cachexia is a multifactorial syndrome characterized by a progressive loss of skeletal muscle mass, along with adipose tissue wasting, systemic inflammation and other metabolic abnormalities leading to functional impairment. Cancer cachexia has long been recognized as a direct cause of complications in cancer patients, reducing quality of life and worsening disease outcomes. Some related conditions, like sarcopenia (age‐related muscle wasting), anorexia (appetite loss) and asthenia (reduced muscular strength and fatigue), share some key features with cancer cachexia, such as weakness and systemic inflammation. Understanding the interplay and the differences between these conditions is critical to advance basic and translational research in this field, improving the accuracy of diagnosis and contributing to finally achieve effective therapies for affected patients.

## 1 Introduction

Coming from the Greek words ‘kakos’ and ‘hexis’, cachexia means ‘bad condition’ and has been clinically described as long as 2000 years ago by Hippocrates.^1^, ^2^, ^3^, ^4^, ^5^, ^6^, ^7^ Cachexia is a multifactorial syndrome associated with numerous chronic or end stage diseases, such as cancer, acquired immunodeficiency syndrome (AIDS), congestive heart failure, chronic obstructive pulmonary disease, rheumatoid arthritis and tuberculosis among others.^2^, ^5^, ^7^, ^8^, ^9^, ^10^, ^11^

Cachexia is a complex systemic disease, involving several metabolic pathways in different tissues and organs, and is characterized by systemic inflammation, progressive weight loss and depletion of adipose tissue and skeletal muscle that cannot be fully reversed by conventional nutritional support.^2^, ^3^, ^7^, ^12^, ^13^, ^14^, ^15^, ^16^

Metabolically, there is resistance to anabolic signals, an overall catabolic state and a negative energy balance.^15^ Anorexia, asthenia, sarcopenia and anaemia are also involved in the clinical features of cachexia, contributing to further reduce quality of life.^2^, ^3^, ^17^ Although weight loss is a key feature of cachexia, it is important to emphasize that its wasting process is remarkably different from starvation‐associated wasting.^15^ Unlike starvation, where lean mass is preserved and adipose tissue is primarily affected, in cachectic patients, the most important event is the wasting of skeletal muscle, with or without fat loss.^15^, ^18^ In fact, cachexia shares some similarities and clear differences with other syndromes like age‐related loss of muscle mass (sarcopenia), anorexia, malabsorption, hyperthyroidism and starvation.^12^, ^19^

As previously mentioned, cachexia is associated with multiple chronic or end stage conditions and develops through similar pathways, regardless of the primary disease.^2^, ^3^, ^7^, ^8^, ^9^, ^10^, ^11^, ^20^, ^21^ Recently, the wasting process was proposed to follow a specific metabolic pattern, most often associated with advanced stages of the underlying condition, that is characterized by a persistent increase of catabolic turnover and a non‐compensatory anabolic activity.^21^

Cancer‐associated cachexia has been the most studied and is the best characterized. Cachexia occurs in up to 80% of cancer patients and is recognized as a direct cause of reduced quality of life, contributing to at least 20% of cancer‐associated deaths and limiting therapeutic options for cancer patients.^5^, ^15^, ^22^, ^23^, ^24^ It is also associated with high costs concerning the healthcare.^25^, ^26^

The specific aetiology and causes of cachexia are complex and only partially understood.^12^, ^27^, ^28^ Consequently, it is very difficult to assess cachexia objectively, particularly in its initial phase.^15^, ^29^, ^30^

It is essential to understand the pathophysiological basis of cancer cachexia and to be able to distinguish it from other related syndromes: only so will we be able to establish an early and accurate diagnosis and adopt timely therapeutic measures.^3^, ^7^, ^12^, ^29^, ^31^, ^32^, ^33^

In this review, we address these issues, bringing together recent data concerning the molecular signalling pathways involved in cachexia, particularly those that may offer therapeutic opportunities. Similarities and differences with other related syndromes — sarcopenia, anorexia and asthenia — are also discussed.

## 2 Cancer cachexia

Several research teams have proposed definitions of cachexia and tried to establish criteria for an accurate and timely diagnosis.^2^, ^12^, ^27^, ^34^, ^35^, ^36^ Different frameworks have been proposed, including a generic approach for cachexia associated with any underlying disease and frameworks to specifically assess cancer cachexia.^2^, ^12^, ^37^, ^38^, ^39^ In 2011, Fearon et *al*. proposed the most accepted framework for diagnosing cancer cachexia.^2^ This approach is based on three key features: a weight loss >5% over past 6 months (in the absence of simple starvation), a body mass index <20 and any degree of weight loss >2%, or an appendicular skeletal muscle index consistent with sarcopenia (male patients <7.26kg/m^2^, female patients <5,45kg/m^2^) and any degree of weight loss >2%.^2^ Because cancer cachexia can co‐occur with obesity, fluid retention and large tumours, all of which can mask weight loss, this framework also recommends a direct measure of muscularity.^2^, ^40^

The same framework also defined three stages in cancer cachexia, namely pre‐cachexia, cachexia and refractory cachexia.^2^, ^30^ An early diagnosis is much more efficient at preserving the patient's quality of life than a late one.^30^, ^41^

The presence of cancer cachexia does not depend on the tumour size.^42^ In fact, the incidence of cancer cachexia varies with the tumour type: in gastric or pancreatic cancer patients, the incidence is over 80%; nearly 50% of patients with lung, prostate, or colon cancer are affected and approximately 40% of patients with breast tumours, advanced head and neck and some leukaemias develop the syndrome.^3^, ^43^, ^44^, ^45^ Furthermore, within a given type of cancer, the degree and extension of cachexia depend on tumour stage.^13^, ^27^, ^46^

The pancreatic cancer is the tumour type in which cancer cachexia is more frequent.^47^ In fact, the pancreatic insufficiency may be previous to the tumour emergence.^47^ The loss of insulin production can constitute an additional problem that aggravates cachexia.^47^ The imbalance between insulin production and secretion leads to a loss of the anabolic function of insulin and its metabolic control in all cachectic patients.^15^, ^47^ Indeed, several cancer patients present insulin resistance that may be promoted by the tumour.^15^ This resistance became gradually worst during the cancer cachexia development and promotes muscle wasting.^15^

Importantly, chemotherapy and radiotherapy may contribute to this cachectic syndrome.^13^ Chemotherapy exacerbates cancer cachexia, because some chemotherapeutic agents induce pro‐cachectic molecules and can up‐regulate muscle wasting.^19^, ^48^, ^49^, ^50^ Cancer treatment based on platinum was described to be associated with weight loss, fatigue and inflammation in cancer patients, because it induces pro‐cachectic cytokines and myostatin, leading to muscle wasting.^48^ For instance, cisplatin is able to regulate muscle wasting through the activation of the nuclear factor‐kappa B (NF‐κB) signalling pathway, which has been convincingly associated with cachexia.^48^, ^49^ For the first time, Pin *et al*. demonstrated that cancer‐induced and chemotherapy‐induced cachexia are characterized by a few shared metabolic abnormalities.^19^

### 2.1 Inflammation

Inflammation is a major driver of cachexia, affecting the function of several tissues including skeletal muscle, fat, brain and liver.^14^, ^15^ In 1985, Cerami's group proved that circulating mediators could cause cachexia, identifying tumour necrosis factor alpha (TNF‐α), initially termed ‘cachectin’.^51^, ^52^ In cancer cachexia, pro‐inflammatory cytokines produced by immune cells and tumour cells most prominently TNF‐α; interleukin‐1, ‐6 and ‐8 (IL‐1, IL‐6 and IL‐8); and interferon gamma (IFN_γ_) help driving the wasting phenotype associated with this syndrome and are classified as procachetic factors by Argilés and Lopez‐Soriano.^3^, ^15^, ^53^, ^54^, ^55^

TNF‐α has a direct catabolic effect on skeletal muscle, by activating the NF‐κB pathway and inducing ubiquitin‐mediated proteasome degradation (UPR) of muscle protein.^56^, ^57^, ^58^, ^59^ TNF‐α is largely responsible for increased gluconeogenesis, proteolysis and loss of adipose tissue in cachectic patients and is associated with up‐regulation of uncoupling proteins (UCPs) 2 and 3 in cachectic skeletal muscle.^28^, ^56^, ^60^, ^61^, ^62^ Additionally, TNF‐α promotes the accumulation of neutrophils and macrophages in skeletal muscle.^63^ The increase of neutrophils and neutrophil infiltration in the tumour are associated with poor outcomes and more severe manifestations of cachexia.^64^ Furthermore, neutrophil‐derived proteases and angiotensin II may trigger a cascade of events required for the progression toward cachexia.^65^ Curiously, it seems that membrane‐bound cathepsin G expressed on neutrophils may generate angiotensin II from angiotensin I and angiotensinogen.^66^ Furthermore, elevated levels of angiotensin II in plasma may cause muscle protein degradation and inhibition of protein synthesis and thus promote cancer cachexia.^65^, ^67^, ^68^

The association of IFN_γ_ with cachexia is still not clear, but studies have shown that IFN_γ_ can synergize with TNF‐α to promote muscle wasting.^15^, ^58^, ^69^, ^70^ IFN_γ_ is also an inhibitor of myosin mRNA in skeletal muscle cells, and it is able to activate ubiquitin gene expression.^61^, ^69^, ^71^

Serum concentrations of IL‐1 increase in cachectic patients, but its role in tissue wasting remains a matter of debate.^58^, ^72^ On the one hand, IL‐1 is thought to induce anorexia in cachectic patients by increasing tryptophan plasma concentrations, leading to increased serotonin levels and causing early satiety and suppression of appetite.^15^, ^28^, ^71^, ^73^ Opposite results from other studies show that high circulating IL‐1 failed to affect food intake or weight loss, suggesting that IL‐1 may have a local tissue‐specific effect or must be present in high pharmacologic doses to produce cachectic response.^15^, ^74^

The pro‐inflammatory cytokine IL‐6 is found in high levels in cachectic patients and is correlated with weight loss.^28^, ^61^, ^75^ IL‐6 was associated with cachexia in rodent models^58^, ^61^, ^76^ and is thought to induce the activation of inflammatory and catabolic pathways, resulting in the suppression of protein synthesis in muscle cells by Janus kinase (JAK) signalling.^58^, ^61^, ^75^, ^76^, ^77^ Inflammation by IL‐6 also induces lipolysis and the browning of the white adipocytes, apparently through up‐regulation of UCP1.^3^, ^78^, ^79^

Elevated serum IL‐8 level was associated with weight loss and was significantly correlated with cachexia in pancreatic cancer patients.^54^, ^55^, ^80^ In gastric cancer patients, an IL‐8 genetic polymorphism was associated with the onset and development cachexia.^55^, ^80^

Decreased expression of anti‐inflammatory cytokines such as IL‐4, IL‐10 and IL‐12 accompanies the up‐regulation of pro‐inflammatory cytokines, further disrupting the balance between pro‐ and anti‐inflammatory stimuli.^15^, ^58^

### 2.2 Skeletal and cardiac muscle wasting

**Figure 1:**
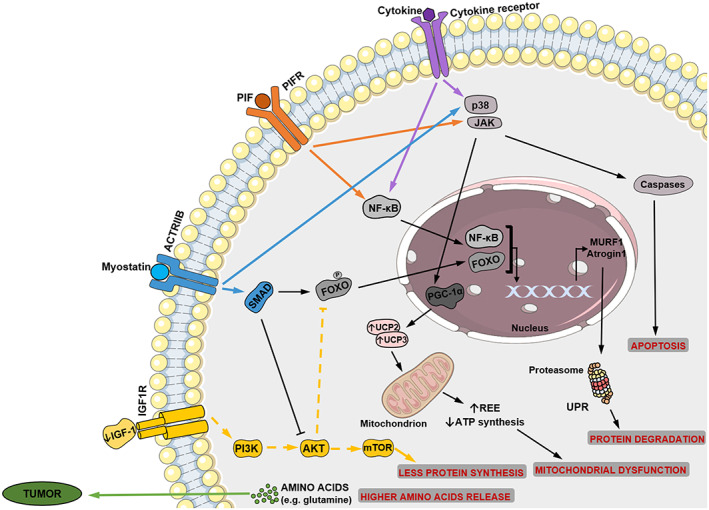
Molecular signalling involved in muscle wasting during cancer cachexia. Inflammatory mediators, such as pro‐inflammatory cytokines (interleukin‐1 and tumour necrosis factor‐α) and myostatin, and proteolysis‐inducing factor (PIF), derived from the tumour and/or immune cells, activate intracellular signals. Cytokines and PIF, through nuclear factor‐kappa B (NF‐κB), activate forkhead box O (FOXO) leading to increased transcription of ubiquitin ligase genes—Atrogin 1 and muscle RING finger‐containing protein 1 (MURF1)—that promote muscle protein degradation. The activation of p38 and Janus kinase/mitogen‐activated protein kinase (JAK/MAPK) cascades by PIF, cytokines and myostatin, leads to apoptosis mediated by caspases. Myostatin can also activate protein degradation through FOXOs. Additionally, myostatin may decrease protein synthesis, inhibiting protein kinase B (AKT) through SMAD. Insulin‐like growth factor‐1 (IGF‐1) is decreased during muscle wasting, suppressing the IGF‐1 pathway (dashed lines) and therefore inhibiting protein synthesis. Peroxisome proliferator‐activated receptor‐γ co‐activator 1α (PGC1α) increases uncoupling protein (UCP) expression, leading to mitochondrial dysfunction. The consumption of high levels of amino acids, such as glutamine, by the tumour increases protein breakdown in skeletal muscle, contributing to cancer cachexia. PIFR, PIF receptor; ACTRIIB, activin receptor type IIB; IGF1R, insulin‐like growth factor‐1 receptor; PI3K, phosphatidylinositol 3‐kinase; mTOR, mammalian target of rapamycin; UPR, ubiquitin‐mediated proteasome degradation; REE, resting energy expenditure

**Figure 2:**
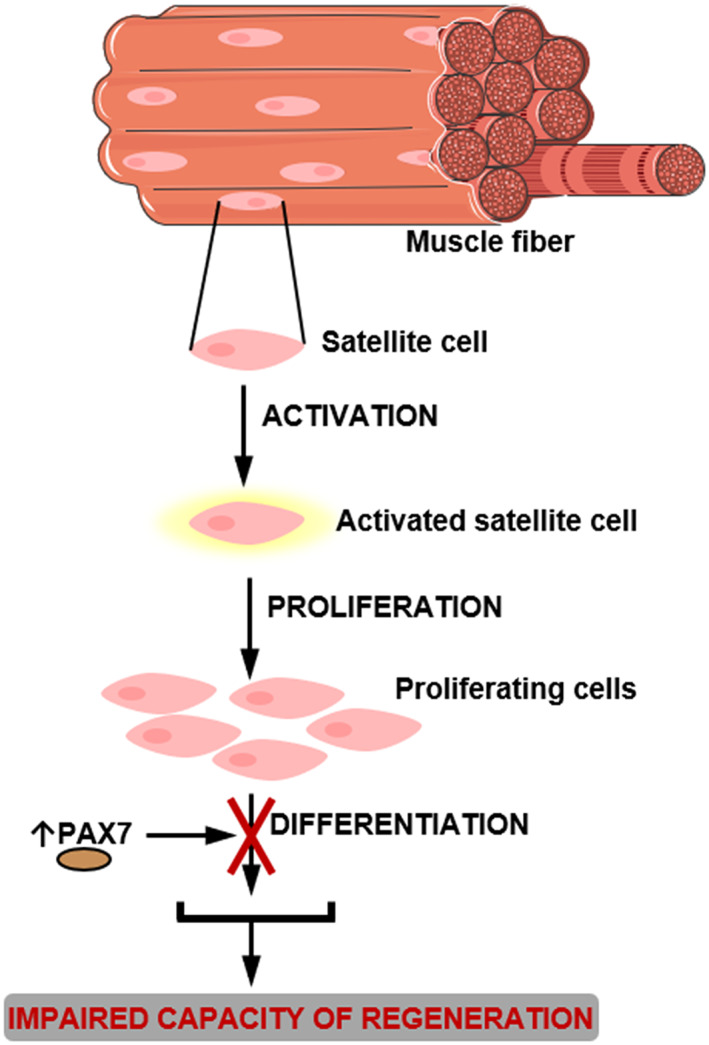
Impaired regeneration capacity during cancer cachexia. Satellite cells are dysregulated during cancer cachexia: although they are able to be activated and proliferate, they cannot complete their differentiation process, because of persistent expression of Paired box 7 (PAX7), via nuclear factor‐kappa B (NF‐κB) activation. PAX7 negatively regulates MyoD and myogenin, which mediate differentiation

The loss of skeletal muscle tissue is a key feature of cancer cachexia and its best studied aspect (*Figure* 1).^3^, ^4^, ^23^ The muscles are a source of amino acids that may be released for energy production during catabolic processes.^81^ Muscle homeostasis is maintained by a balance between the synthesis and degradation of muscle protein.^15^ However, when excessive protein degradation and/or decreased protein synthesis occurs in skeletal muscle, the imbalance can cause muscle wasting and cachexia.^4^, ^15^, ^81^ Skeletal muscle wasting involves several molecular alterations (*Figures* 1 and 2), all of which are associated with inflammation, protein metabolism, apoptosis and decreased tissue regeneration.^3^

There are three main pathways associated with protein degradation described in skeletal muscle: the UPR pathway, the autophagy pathway and calcium‐activated protease calpains.^15^, ^82^

The UPR system is specifically up‐regulated by skeletal muscle cells during cancer cachexia through the expression of ubiquitin‐ligases: the muscle RING finger‐containing protein 1 (MURF1) and Atrogin‐1.^15^, ^69^, ^82^, ^83^ The expression of these ligases involved in muscular proteolysis and muscle wasting is increased by the activation of forkhead box O (FOXO) family transcription factors.^3^, ^15^ FOXO activation occurs via NF‐κB signalling, which is activated by cytokines such as TNF‐α and IL‐1 and by proteolysis‐inducing factor (PIF).^3^, ^15^, ^84^

Importantly, the p38 and JAK/mitogen‐activated protein kinase (MAPK) cascades may also be activated by the same cytokines and PIF, activating caspases and, consequently, apoptosis.^3^ Myostatin, which acts through activin receptor type IIB (ACTRIIB)‐mediated signalling, can also activate protein degradation by the FOXO and can trigger apoptosis via MAPK cascade.^3^

In the last 15 years, autophagy has also been recognized as a main promoter of proteolysis in skeletal muscle and as an important player in cancer cachexia.^3^, ^15^, ^82^, ^85^ Calcium‐activated protease calpains have also been associated with initiation of protein breakdown during cachexia.^15^, ^82^, ^86^

In parallel with the activation of catabolic pathways, there is also inhibition of anabolic pathways contributing to muscle wasting during cancer cachexia.^15^ Insulin‐like growth factor‐1 (IGF‐1) generally activates insulin‐like growth factor‐1 receptor (IGF1R)/phosphatidylinositol 3‐kinase (PI3K)/protein kinase B (AKT)/mammalian target of rapamycin (mTOR) signalling, promoting protein synthesis and repressing protein degradation (via FOXO phosphorylation).^3^ Interestingly, both cancer patients and mouse models of cancer cachexia present low circulating levels of IGF‐1.^3^, ^81^, ^87^ In fact, during muscle wasting, IGF‐1 is decreased, effectively suppressing protein synthesis.^3^, ^4^ Additionally, the insulin resistance present in this condition promotes the muscle wasting too.^15^ Myostatin can also decrease protein synthesis through the activation of the SMAD complex and consequent repression of AKT.^3^, ^88^ In addition, myostatin decreases myoblast proliferation because of its inhibition of the myogenic programme.^3^, ^88^

Normally**,** the skeletal muscle regenerates by the activation and differentiation of specific stem cells, known as satellite cells.^15^, ^89^ However, in murine cancer models and in patients with cancer cachexia, these cells are dysregulated.^4^, ^15^, ^89^ Although satellite cells can still be activated and proliferate, they cannot differentiate into skeletal muscle cells because of a persistent expression of the self‐renewing factor Paired box 7 (Pax7) mediated by NF‐κB (*Figure* 2).^15^, ^89^ Pax7 negatively acts on MyoD and myogenin expression, essential mediators of differentiation, impairing the regeneration process.^89^

PIF can also increase protein degradation through the UPR pathway and inhibit protein synthesis by phosphorylating eukaryotic translation initiation factor 2α (eIF2α).^3^, ^50^, ^84^, ^90^

Moreover, the down‐regulation of protein synthesis and stimulation of protein degradation may result from a decreased availability of amino acids.^82^ Cancer cells require amino acids, namely glutamine, resulting in low circulating glutamine levels and breakdown of skeletal muscle protein to release amino acids from muscle cells.^3^, ^87^, ^91^ Thus, glutamine supplementation has been suggested to attenuate the muscle wasting in cancer patients.^92^

Cachectic muscles also show impaired mitochondrial metabolism.^3^, ^15^, ^50^ During cachexia, skeletal muscle mitochondria presents an ineffective ATP synthesis, because the proton gradient that drives this synthesis is disrupted by activation of UCPs (UCP2 and UCP3),^3^, ^15^, ^93^ causing a general increase of resting energy expenditure described in cachectic patients.^3^, ^15^, ^93^ The increased expression of UCPs may be the result of cytokine‐induced stabilization and activation of peroxisome proliferator‐activated receptor‐γ co‐activator 1α (PGC‐1α).^3^, ^50^, ^94^

As with skeletal muscle, the cardiac muscle is an important target in cancer cachexia and the loss of body weight is often accompanied by wasting of cardiac muscle.^13^, ^15^, ^95^, ^96^ Identical observations were made in laboratory rodents bearing cachexia‐inducing tumours, where cardiac atrophy is frequently observed.^97^ In the mouse colon‐26 cancer cachexia model, cardiac alterations include marked fibrosis, disrupted myocardial ultrastructure and altered composition of contractile proteins (e.g. troponin I and myosin heavy chain).^98^

As with skeletal muscle, pro‐inflammatory cytokines play a major role in mediating cardiac dysfunction and NF‐κB inhibition protected tumour bearing mice against cachexia.^13^, ^15^, ^99^ Proteolysis of cardiac muscle is mediated by the UPR, and cachectic tumour‐bearing mice show high levels of protein ubiquitylation and up‐regulated MURF1 and atrogin‐1 expression.^13^, ^98^ Furthermore, in mice with cancer cachexia, cardiac atrophy has been associated with growth inhibition through the activation of ACTRIIB signalling mediated by a subset of TGFβ family ligands including myostatin, activin and growth/differentiation factor 11 (GDF11).^100^

### 2.3 Adipose tissue: lipolysis and browning

**Figure 3:**
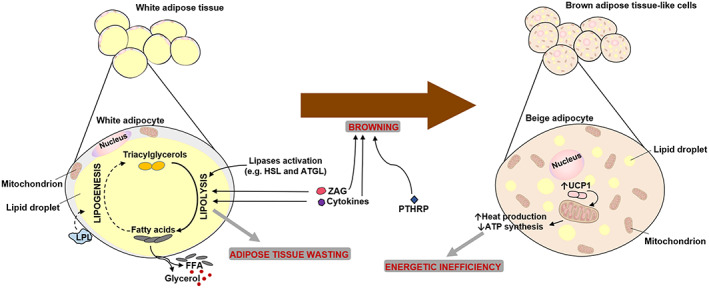
Adipose tissue lipolysis and browning during cancer cachexia. In cancer cachexia, adipose tissue wasting is observed. High levels of circulating free fatty acids (FFA) and glycerol are observed, because of a massive lipolysis in white adipose tissue (WAT), promoted by lipases activation, zinc‐α2‐glycoprotein (ZAG) and cytokines. Additionally, these high levels of FFA may also result from lipoprotein lipase (LPL) decreased activity that reduces lipogenesis (inhibition represented by the dashed line). Moreover, WAT can acquire features of brown adipose cells, a process called ‘WAT browning’. In these beige adipocytes, uncoupling protein 1 (UCP1) is expressed, promoting uncoupling mitochondrial respiration. This results in heat production and less ATP synthesis, leading to an energetic inefficiency. This browning can be promoted by cytokines, ZAG and tumoural‐derived compounds such as parathyroid‐hormone‐related protein (PTHRP). HSL, hormone‐sensitive lipase; ATGL, adipose triglycerides lipase

The importance of pathologic changes in adipose tissue has been increasingly recognized in recent years, and some of those changes are involved in cachexia (*Figure* 3).^13^, ^15^, ^50^, ^79^ Histologically, there are two types of adipose tissue: white adipose tissue (WAT), which is mostly involved in energy storage in the form of triglycerides, and brown adipose tissue (BAT) involved in thermoregulation.^79^, ^101^, ^102^, ^103^, ^104^ Until recently, BAT was thought to be functionally present only on neonates, but in fact is also present in adults.^79^

Cachectic patients often show high levels of circulating free fatty acids and glycerol resulting from a massive lipolysis in WAT through the activation of lipases like hormone‐sensitive lipase and adipose triglycerides lipase.^3^, ^13^, ^15^, ^105^, ^106^, ^107^ IL‐6, TNF‐α and zinc‐α2‐glycoprotein (ZAG, also called lipid‐mobilizing factor) are able to promote lipolysis in cancer cachexia.^78^, ^79^, ^84^, ^106^, ^108^ Additionally, inhibition of adipogenesis resulting from down‐regulation of adipogenic factors and reduced lipogenesis because of decreased activity of lipoprotein lipase (LPL) may also contribute to WAT wasting during cancer cachexia.^3^, ^79^, ^106^, ^109^, ^110^

During cancer cachexia, WAT cells may acquire some of the features that characterize BAT cells (‘beige adipocytes’),^3^, ^50^ a process known as ‘WAT browning’ that takes place during the initial steps of cancer cachexia, before muscle wasting.^13^, ^70^, ^101^, ^105^, ^111^ BAT is an active tissue associated with hypermetabolism because of the presence of UCP1.^13^ When the switch from WAT to BAT‐like cells (beige adipocytes) occurs, UCP1 expression is increased and promotes uncoupling mitochondrial respiration towards thermogenesis rather than ATP synthesis, leading to heat production and energetic inefficiency.^13^, ^50^, ^70^, ^102^ Thus, WAT browning contributes to increased systemic energy expenditure and, therefore, to cancer cachexia.^13^, ^70^ WAT browning can be triggered by pro‐inflammatory mediators, such IL‐6, ZAG, or by tumour‐derived compounds like parathyroid‐hormone‐related protein (PTHRP).^50^, ^70^, ^79^, ^112^, ^113^

### 2.4 Liver

The liver undergoes multiple metabolic and histological changes during cancer cachexia that help driving tissue wasting and also reflect hepatic overload and damage.^13^, ^114^, ^115^, ^116^

In the context of cancer, the liver often contributes to amplify systemic inflammation by producing acute phase proteins, which may help driving the breakdown of muscle proteins into amino acids.^13^, ^15^, ^114^, ^116^ It occurs an increase in the hepatic production of glucose and in the Cori Cycle activity.^15^, ^26^, ^117^ Protein breakdown produces a flow of nitrogen in form of amino acids, mainly alanine into the liver.^3^, ^13^ Hepatic gluconeogenesis is then able to use amino acids, glycerol (from adipose tissue), and lactate to produce glucose.^3^, ^15^ However, this pathway is extremely demanding, contributing to energetic inefficiency and a negative balance, promoting hepatic damage and weight loss.^3^, ^5^, ^15^, ^118^ In fact, hepatomegally has been described in cachectic patients in association with increased IL‐6 circulating levels and hepatic metabolic overload.^15^, ^114^, ^115^, ^119^ Inflammation and steatosis are typical features of hepatic damage associated with and cachexia.^15^, ^78^, ^120^, ^121^

### 2.5 Brain

In cancer patients, the brain areas that control energy homeostasis may suffer from functional alterations that may contribute to the onset and progression of some features of cancer cachexia, including anorexia, and increased catabolism in muscles and adipose tissue.^50^, ^122^, ^123^ Neuroinflammation seems to accompany tumour growth and to contribute to cancer cachexia, because hypothalamic inflammation can act both as receiver and as amplifier of systemic inflammation.^50^, ^122^, ^123^ Inflammation increases serotonin availability in the hypothalamus.^13^, ^122^, ^124^, ^125^ The alteration of serotonin levels was associated with changes in food intake of cachectic tumour‐bearing mice.^124^ Using murine‐derived neuropeptide‐Y (NPY)‐secreting hypothalamic cell lines, it was described that the serotonin can reduce the secretion levels of hypothalamic NPY, leading to a decrease of food intake.^122^, ^124^

Hypothalamic inflammation with increased IL‐1β levels also triggers the hypothalamic‐pituitary‐adrenal axis, resulting in increased release of glucocorticoids that promote the breakdown of adipose tissue and skeletal muscle, directly causing cachexia.^50^, ^122^, ^123^

### 2.6 The gastrointestinal tract and the gut microbiota

In cancer patients, altered intestinal permeability may occur because of the cytotoxic agents used in cancer therapies.^126^ These intestinal permeability alterations together with translocation of bacteria or bacterial components (e.g. lipopolysaccharide) may also contribute to systemic inflammation and, ultimately, to cachexia.^13^, ^15^, ^127^ Gut barrier dysfunction is a syndrome characterized by dysruption and leakage of the gut epithelial barrier.^13^, ^128^ Experimental data from a mouse model of colon cancer cachexia show that dysruption of the gut barrier accompanied tumour growth, leading to endotoxemia and systemic inflammation.^128^ Gut barrier dysfunction is also associated with malabsorption of nutrients, further contributing to the energy imbalance observed in cancer cachectic patients.^13^

Changes in the gut microbiota may also play a role in cancer cachexia, as different bacterial strains have different abilities to produce nutrients and pro‐inflammatory molecules that may induce muscle and adipose tissue wasting.^13^, ^129^ Experimental data from mouse models support this hypothesis and help associating alterations in the gut microbiome with cancer cachexia.^130^, ^131^

Ghrelin, a peptide that is mainly produced in the stomach and increases appetite, stimulating hunger and food intake, is highly increased in patients with cancer cachexia.^3^, ^13^, ^132^, ^133^, ^134^, ^135^ Ghrelin can also directly affect skeletal muscle cells, inhibiting protein degradation induced by cytokines.^3^, ^136^, ^137^, ^138^, ^139^ In this context, increased ghrelin levels are likely to be a compensatory mechanism to buffer cachexia and to represent a mechanism to counter‐balance anorexia.^3^, ^13^, ^15^

### 2.7 Therapeutic options

The more effective way to treat cancer cachexia is to cure the underlying condition, i.e., cancer.^140^, ^141^ However, curing many types of cancer remains an unmet challenge.^140^, ^141^ Therefore, therapeutics options for cancer cachexia often aim to treat and ameliorate cachexia itself.^3^, ^12^, ^141^ Unfortunately, the therapeutic options for this syndrome are limited and not always effective.^3^, ^12^ Cancer cachexia cannot be totally reversed or prevented by using conventional nutritional support or even total parenteral nutrition.^7^, ^12^, ^13^

Several therapeutic interventions with multiple agents have been described and tested.^7^, ^32^, ^140^, ^142^ Supplementation with omega‐3 fatty acids that reduce IL‐1 and TNF‐α production and improve the efficacy of nutritional support has been tested.^7^, ^140^, ^141^ Glucocorticoids have also been used in order to mitigate some symptoms in patients, by inhibiting the synthesis/release of pro‐inflammatory cytokines and enhancing NPY levels, and show a rapid onset of effect on appetite.^7^, ^140^, ^141^ Non‐steroidal anti‐inflammatory drugs and drugs leading to cytokine inhibition have also been hypothesized to have a promising positive effect in cancer cachexia, by diminishing the inflammatory status and improving muscle wasting.^7^, ^50^, ^140^, ^141^ Amino acid supplementation could also be an approach to consider (like glutamine supplementation that may attenuate muscle wasting in cancer patients).^15^, ^92^, ^143^ The use of megestrol acetate also shows some good results in cancer cachectic patients.^30^, ^41^ Other agents causing an appetite stimulation (like cannabinoids or erythropoietin), antidopaminergics (like metoclopramide), or muscle synthesis stimulation (as branched‐chain amino acids) have been described as possible therapeutic approaches too.^7^, ^140^, ^144^

Additionally, physical activity seems to be an important element for the treatment of cancer cachexia.^32^, ^50^, ^145^, ^146^ Exercise training programs (such as strength and/or aerobic training) demonstrated to have a very clear anti‐inflammatory effect, decreasing pro‐inflammatory cytokines and increasing anti‐inflammatory cytokines.^146^ Exercise may act as a promoter of metabolic and inflammatory pathways disruption during cancer cachexia, improving functional status, body composition and longevity.^32^, ^146^ Nonetheless, additional studies are needed to determine the effectiveness and safety of exercise in cancer cachexia patients.^32^

Because cancer cachexia has a multidimensional background, multimodal therapeutic approaches are a preferred strategy, as a single approach is unlikely to be effective.^3^, ^141^, ^142^, ^147^ These multimodal approaches consist of combinations of interventions, including not only pharmacological drugs and/or nutritional supplementation, but also a program of moderate physical exercise.^13^, ^32^, ^141^, ^142^

## 3 Sarcopenia

Advancing adult age is associated with severe changes in body composition, which affect skeletal muscle mass most severely and may lead to decreased muscle strength and functionality.^148^, ^149^ Irwin Rosenberg proposed the term ‘sarcopenia’ to specifically name this condition characterized by age‐related decrease in muscle mass, with increased risk of physical disability and poor quality of life.^148^, ^150^, ^151^, ^152^ The pathophysiology of sarcopenia is complex, and multiple mechanisms contribute to its development including a down‐regulation of anabolic hormones like insulin, sexual steroids and growth hormone, an increased apoptotic activity in muscle cells and increased circulating pro‐inflammatory cytokines.^35^, ^153^, ^154^, ^155^

Aging is associated with a chronic state of systemic low‐grade inflammation, characterized by increased plasma levels of pro‐inflammatory mediators like TNF‐α and IL‐6, which are able to stimulate proteolysis mainly via UPR, as previously described for cancer cachexia.^154^, ^156^, ^157^, ^158^, ^159^, ^160^, ^161^, ^162^ However, the role of UPR in sarcopenia is still contentious.^163^, ^164^, ^165^ Some studies have described an up‐regulation of components of the UPR in sarcopenia while other have shown a down‐regulation or no differences.^166^, ^167^, ^168^, ^169^, ^170^

Enhanced production of reactive oxygen species is thought to promote sarcopenia by destabilizing mitochondria in skeletal muscle fibres and thus increasing their susceptibility to apoptotic stimuli while also down‐regulating pathways associated with mitochondrial biogenesis.^157^, ^163^, ^171^ Oxidative stress may also promote other sarcopenia‐associated processes, such as proteolysis, up‐regulation of TNF‐α levels and inhibition of muscle cell differentiation.^163^, ^171^, ^172^, ^173^

In fact, the apoptosis of satellite cells contributes to the reduction of muscle mass and function associated with age.^174^, ^175^ During aging, the regenerative potential of skeletal muscle is lost, because of impairment of satellite cells, which lose their capacity to regenerate and to self‐renewal.^174^, ^175^

Additionally, a loss of motoneurons, neuromuscular remodelling, denervation and fibre‐type switch may also contribute to reduced numbers of muscle fibres and the overall muscle mass.^155^, ^163^, ^176^, ^177^, ^178^

Dietary changes leading to lower and/or deficient intake of energy and protein may contribute to loss of muscle mass and function.^35^ Immobility and reduced physical inactivity may also contribute to the onset of sarcopenia in older people.^35^, ^148^

Finally, cancer cachexia (often occurring in older patients) may co‐occur with sarcopenia.^35^, ^148^ In fact, these two syndromes overlap considerably, especially in older patients.^35^, ^155^ Most cachectic individuals are also sarcopenic; however, most sarcopenic individuals are not considered as being cachectic, so sarcopenia can be considered as a component of cachexia.^12^, ^35^, ^148^, ^179^ Sarcopenia is not associated with weight loss, in opposition to cachexia that is associated with significant weight loss, that leads to a reduction of both fat and fat‐free mass.^12^, ^180^ Furthermore, although both processes are associated with systemic inflammation, cachexia is characterized by more intense inflammatory processes in contrast with sarcopenic patients that often show a slight or undetectable systemic inflammation.^35^, ^155^, ^180^, ^181^ Sarcopenia is mediated by a number of factors, while in cachexia, the activation of proinflammatory cytokines has a major and direct effect on disrupting muscle metabolism.^155^

To help clarify the diagnosis of sarcopenia, the European Working Group on Sarcopenia in Older People (EWGSOP) initially recommended the use of two criteria, namely low muscle mass and low muscle function (strength or performance).^148^

A recent update by EWGSOP2 designated low muscle strength as the primary criterion for diagnosing sarcopenia. Presently, muscle strength is the most reliable measure of muscle function.^182^ Presence of low muscle strength (criterion 1 – grip strength <30kg or <20kg for men and women, respectively) supports a diagnosis of probable sarcopenia. Additional documentation of criterion 2, low muscle quantity or quality (appendicular lean mass adjusted for height: ALM/height^2^ <7.23 or <5.67kg/m^2^ for men and women, respectively) can confirm the diagnosis of sarcopenia. If criteria 1 and 2 are present together with low physical performance (considered a third criterion 3 – gait speed ≤0.8m/s), sarcopenia is considered severe.^182^

Multiple groups published different operational criteria to define sarcopenia.^183^, ^184^, ^185^, ^186^ In 2011, the International Working Group (IWG) proposed a definition of sarcopenia that includes low physical performance (gait speed <1.0m/s) and low muscle mass (ALM/height^2^ ≤7.23 or ≤5.67kg/m^2^ for men and women, respectively).^184^ The Foundation for the National Institutes of Health (FNIH) Sarcopenia Project presented distinct criteria to define sarcopenia^185^ on the basis of low appendicular lean mass adjusted for body mass index (ALM/BMI <0.789 for men or <0.512 for women), in the presence of low muscle strength (grip strength <26 kg for men or <16 kg for women).^185^ The FNIH definition is more restrictive than the EWGSOP and IWG criteria, which may result in sarcopenia being diagnosed less frequently.^183^

The Asian Working Group for Sarcopenia (AWGS) agreed with the low muscle mass plus low muscle strength and/or low physical performance criteria.^187^ The AWGS recommended cutoff values for muscle mass measurements (<7.0kg/m^2^ for men and <5.4kg/m^2^ or 5.7kg/m^2^ for women, using X‐ray absorptiometry or bioimpedance analysis, respectively), grip strength (<26kg for men and <18kg for women) and gait speed (<0.8m/s).^187^ Of all these definitions, the most widely used criteria for diagnosing sarcopenia is the one proposed by EWGSOP/EWGSOP2.^148^, ^182^

Sarcopenia can also be classified according to its causes and by disease stages.^148^, ^182^, ^188^ Sarcopenia is considered primary or age‐related when no other specific cause is evident but aging itself, or secondary when causal factors other than (or in addition to) aging are evident, such as disease, inactivity, or poor nutrition.^148^, ^182^ The EWGSOP also proposed a sarcopenia staging scheme to help guide the clinical management of this syndrome, defining the stages of ‘presarcopenia’ characterized by low muscle mass without impact on muscle strength or performance, ‘sarcopenia’ with low muscle mass plus low muscle strength or low physical performance and ‘severe sarcopenia’, when all the three criteria meet.^148^

Regarding the possible therapeutic options for sarcopenia, the primary intervention should be physical exercise.^155^, ^189^, ^190^ The advantages of exercise have been demonstrated convincingly and resistance exercise training was shown to increase muscle mass and strength, improving muscle protein accretion.^190^, ^191^ It has been suggested that a combination of several exercises into a programme may achieve better results than a highly focused exercise regimen.^189^, ^190^, ^192^

Nutrition is another important component to consider.^190^, ^192^ However, clinical trials using standardized interventions with single or complex nutritional supplementation are still missing.^190^, ^192^ The use of protein (like some milk‐based proteins) or essential amino acid supplementation (particularly leucine) has shown only minor effects on muscle recovery.^192^, ^193^ The efficacy of vitamin D supplementation to ameliorate sarcopenia is still a matter of debate.^190^, ^192^, ^193^ The use of anabolic hormones also continues to show limited efficacy in treating sarcopenia.^155^, ^190^ Overall, no drug is currently approved for sarcopenia treatment and a combined strategy with nutritional supplementation and exercise programs seems to be the most promising approach for sarcopenia.^189^, ^190^, ^193^

More research is necessary and encouraged in the sarcopenia's field in order to prevent or delay adverse outcomes that arouse heavy burden for patients' health, dependence and economics as well for healthcare systems.^35^, ^182^

## 4 Anorexia

Defined as the loss of appetite, anorexia is considered to be an important component of cachexia and also may play a role in the pathogenesis of sarcopenia.^2^, ^23^, ^194^, ^195^, ^196^, ^197^ However, it is important to emphasize that anorexia can be distinct from both syndromes and can occur independently from either.^12^, ^17^, ^196^, ^198^ With anorexia, the wasting of skeletal muscle that characterizes cachexia does not occur.^199^ Also, nutritional supplement cannot replenish the loss of body mass in cachexia, contrary to what happens in anorexia.^199^ Moreover, anorexia alone cannot explain and cause all the metabolic changes that happens during cachexia.^7^, ^84^, ^198^ Anorexia occurs in other conditions such as psychiatric problems, the use of certain medications, depression, aging that leads to an appetite decrease, gastro‐intestinal problems and a variety of alterations in central neurotransmitters.^12^, ^195^, ^200^

Anorexia may be categorized in two categories, in accordance with the alterations of the volitional or nonvolitional control of eating.^201^ If it occurs because of an altered body image perception leading to the volitional refuse of eating, it is defined as primary anorexia (or anorexia nervosa), and if it happens as a consequence of a higher and/or persistent inflammatory response secondary to chronic or acute diseases, it is defined as secondary anorexia (or disease‐specific anorexia).^201^ In anorexia nervosa, the suppression of appetite is because of the psychiatric need to adjust body image to unrealistic models.^201^, ^202^ In fact, a part of patients suffering from anorexia nervosa may not lack appetite.^203^ In secondary anorexia, the suppression of the appetite is because of the function disruption of the neuronal pathways that regulate the physiological eating behaviour.^201^, ^204^

Anorexia nervosa is thought to be triggered by a combination of multiple biological and social factors.^203^ This condition is closely related to social pressure and media exposure and is not easy to treat.^203^

Anorexia of aging includes a combination of physical and social changes associated with aging (e.g. decline in smell in taste, reduced appetite, delayed gastric emptying, dementia, depression, solitude and poverty).^194^, ^205^ Anorexia of aging is thought to develop through two general mechanisms: the reduced drive to eat resulting from lower energy requirements and earlier satiety signals.^205^ Still, the exact mechanisms underlying anorexia of aging are still to be clarified.^205^ It is important to emphasize that this type of anorexia may occur even in otherwise healthy adults.^205^ Furthermore, anorexia of aging can be considered a direct risk factor for weight loss and consequent sarcopenia.^194^

Secondary anorexia is frequently observed in cancer patients and is associated with limited food intake, worse disease outcomes, increased morbidity and mortality and reduced quality of life.^206^, ^207^ Anorexia is present in up to 50% of newly diagnosed cancer patients and is the fourth most common symptom (after pain, fatigue and weakness) in patients with advanced cancer stages.^7^, ^207^, ^208^ Several factors contribute for anorexia in cancer patients: chemotherapy/drugs, depression, constipation, emesis, decreased gastric emptying, dysphagia, mucositis/stomatitis, reduced or altered taste, pain and tumour products and host factors.^17^

Chronic anorexia in wasting syndromes implies that the adaptative feeding response fails.^209^ Tumour‐released substances such as proinflammatory cytokines, lactate, or PTHRP contribute decisively to anorexia.^195^ Many cytokines (IL‐1α, IL‐1β, IL‐6, IL‐8 and TNF‐α) have a known effect on appetite by modulating central nervous system neurotransmitter cascades.^195^, ^199^, ^204^, ^210^, ^211^, ^212^, ^213^, ^214^, ^215^ It was also hypothesized that cancer anorexia may be the end result of alterations in the neurohormonal signals (central and peripheral) that govern appetite.^216^

Leptin is an adipokine that acts on specific hypothalamic receptors, promoting satiety via downstream neuropeptides such as NPY.^140^, ^195^, ^209^ Cytokines such as IL‐1 may induce anorexia by stimulating the expression and release of leptin and by mimicking leptin's hypothalamic effect.^73^, ^140^, ^195^, ^209^, ^213^ Lactate inhibits food intake via hypothalamic activation of the adenosine monophosphate kinase/malonyl‐CoA signalling pathway.^195^, ^217^ Therefore, cancer anorexia is strongly associated with functional damage of the hypothalamic mechanisms that normally control the eating behaviour.^201^, ^218^ Additionally, cancer treatments as chemotherapy and radiation, which cause nauseas and vomiting, may contribute to anorexia.^7^, ^199^

One of the challenges of anorexia assessment in patients is to characterize all the distinct contributors and then provide a correct and multimodal approach to the treatment.^17^ Therefore, in order to accurately diagnose anorexia, it is important to have valid and reliable methods.^207^ The assessment of anorexia is based in visual analogue scales, numerical scales, verbal descriptors, or individual questionnaires, introducing significant subjectivity into the process of diagnosis.^17^ In clinical practice, a ‘yes/no’ questionnaire (‘do you experience a decreased appetite?’) or the anorexia symptom scale of the European Organization for Research and Treatment of Cancer (EORTC) Quality of Life Questionnaire (QLQ)‐C30 (third version)^219^ is most often used.^207^ Lately, two other methods have been proposed for specifically diagnosing anorexia associated with cachexia: the Anorexia/Cachexia Subscale of the Functional Assessment of Anorexia/Cachexia Therapy (FAACT‐A/CS) questionnaire^220^ and the visual analogue scale for appetite, but the validation of the cut‐offs is still lacking.^35^, ^206^, ^207^ Therefore, nowadays, no gold standard exists for diagnosing anorexia in patients with cancer.^207^

Concerning possible pharmacological treatments for anorexia, a number of specific orexigenics have been developed.^195^ Megestrol was approved to treat anorexia in patients with AIDS and was also shown to improve weight gain and ameliorate anorexia in children with cancer.^195^, ^221^ Cannabinoids also seem to increase appetite by enhancing NPY in the hypothalamus.^195^ Ghrelin and related substances also have an impact on food intake by anorexic patients.^195^, ^222^

## 5 Asthenia

Asthenia is a universal feature of advanced malignancy, and it is another prominent and frequent manifestation of advanced cancer.^17^, ^223^ Most advanced cancer patients show a combination of cachexia and asthenia, but they can also occur independently.^7^, ^17^, ^224^ It is important to emphasize that cachexia is not a requisite for asthenia to occur.^224^, ^225^ Asthenia comes from the Greek ‘asthenos’ and means absence of strength, loss of muscle force and muscle weakness.^3^, ^5^, ^223^ It is characterized physically by profound tiredness after usual or small efforts, accompanied by an unpleasant and anticipatory sensation of generalized weakness and fatigue, and loss of muscle strength. Mentally, asthenia is associated with decreased capacity for intellectual work, impaired concentration, loss of memory and emotional lability.^7^, ^17^, ^223^, ^226^

Although some authors may refer to asthenia with the term ‘fatigue’, it is important to emphasize that fatigue is only one dimension and symptom of asthenia.^227^ In fact, fatigue is defined by tiredness or exhaustion as result of physical or mental effort, while asthenia is characterized by tiredness or exhaustion in the absence of physical or mental effort.^228^ Some authors also describe an asthenia‐fatigue syndrome or cancer‐related fatigue (CRF), when asthenia in associated with cancer.^229^, ^230^, ^231^, ^232^

Even though asthenia has a complex pathophysiology, it seems to be mediated by a combination of factors released by the tumour, host responses (cytokines) and direct consequences of the tumour presence.^223^, ^225^, ^229^ The brain (associated with fatigue) and the muscle (associated with weakness) seem to be the two major organs that should be address to understand the mechanisms underlying asthenia.^225^ More studies are necessary to obtain a deeper understanding of asthenia and its mechanisms.

Metabolic abnormalities involved in the development of cachexia also generally lead to the development of asthenia, and the loss of muscle resulting from progressive cachexia provides a rationale for asthenia.^17^, ^224^, ^225^, ^228^ However, it is important to emphasize that muscle wasting does not occur because of asthenia itself.^17^, ^224^ In addition to cachexia, there are numerous other factors that contributing to asthenia in cancer patients including anaemia, infection, muscle abnormalities/immobility, chemotherapy and/or radiotherapy, metabolic problems, cytokines, psychological distress and pain/drug side effects.^17^, ^229^

Asthenia is frequently underdiagnosed or neglected in cancer patients, accepting the fatigue as a ‘normal’ symptom.^229^ However, asthenia and the fatigue associated have an important effect in the life quality of patients.^233^ Even so, because asthenia is a subjective sensation for the patients, its assessment is hard and difficult.^17^, ^223^ The most common approaches to the assessment of asthenia were reviewed by Bruera and Sweeney in 2000.^17^ The first approach is to assess the patient's functional capacity in a standard/normal task such as walking in a treadmill or with simple and daily tasks.^17^ Another approach employs scales to rate the patient's functional abilities and is the most used in oncology.^17^ The most commonly used scales are the Eastern Cooperative Oncology Group – Performance Status (ECOG‐PS) and Karnofsky Performance Status (KPS) scales.^17^, ^234^, ^235^, ^236^ The last approach is the subjective assessment of fatigue employing questionnaires.^17^, ^237^, ^238^ Despite these efforts, it is currently accepted that self‐assessment should be the ‘gold standard’, because asthenia is essentially a subjective sensation.^17^

Berger *et al*., in 2015, presented Standards of Care for CRF Management using the National Comprehensive Cancer Network (NCCN) Clinical Practice Guidelines in Oncology (NCCN Guidelines).^232^ These standards help managing CRF and should give useful guidance for professionals, by proposing a treatment algorithm.^232^

Corticosteroids have been suggested to decrease asthenia in advanced cancer patients,^17^, ^239^ but their long‐term use is associated with important side effects.^17^, ^239^ The use of psychostimulants seems to be effective only in patients with asthenia having an opioid toxicity.^17^ Megestrol acetate was also associated with clinical benefits in asthenic patients.^17^ Importantly, physical exercise, physiotherapy and occupational therapies seem to improve asthenia.^17^, ^239^

## 6 Conclusion

**Figure 4:**
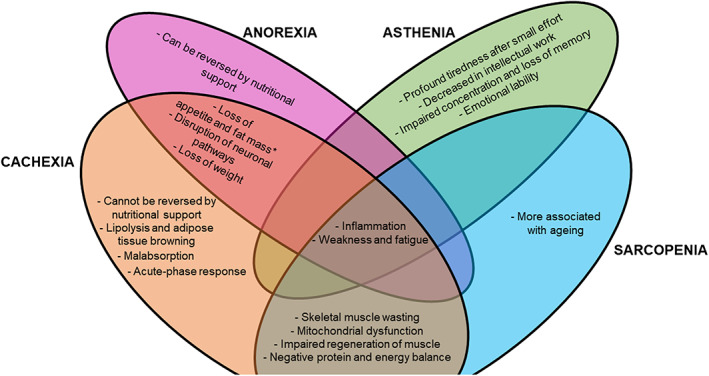
Links and overlaps between related conditions. Inflammation, weakness and fatigue are features of cachexia, sarcopenia, anorexia and asthenia. *Cachexia can occur with or without loss of appetite and fat mass

Although cachexia, sarcopenia, anorexia and asthenia can be defined as distinct clinical conditions, they share multiple important common points and a certain degree of overlap (*Figure* 4 and *Table* 1). The two features that better demonstrate this overlap are inflammation and weakness (*Figure* *4*). These features may be observed not only in cachectic or sarcopenic patients but also in patients suffering from anorexia or asthenia. Because weakness/fatigue and inflammation are features transversal to several conditions, clinicians cannot exclusively use these features to distinguish between the four conditions presented. Clinicians should look for features (or combination of features) that make each condition unique (*Figure* *4*) and to evaluate them in the context of each patient's clinical history and personal profile (e.g. earlier diseases, presence of mental conditions, age).

**Table 1:** Summary of the definitions, features and diagnosis criteria of the four conditions

Condition	Definition	Features	Diagnosis/assessment criteria
Cachexia	Multifactorial syndrome characterized by an ongoing loss of skeletal muscle mass (with or without loss of fat mass) that cannot be fully reversed by conventional nutritional support and to progressive functional impairment^2^	‐ Loss of weight (with or without loss of fat mass) ‐ With or without loss of appetite ‐ Inflammation ‐ Negative protein and energy balance ‐ Skeletal muscle wasting ‐ Lipolysis and browning of adipose tissue ‐ Impaired regeneration of muscle cells ‐ Mitochondrial dysfunction ‐ Disruption of neuronal pathways ‐ Acute‐phase response ‐ Malabsorption 2, 3, 50, 89, 122	‐ Weight loss >5% over past 6 months (in absence of simple starvation); or ‐ Body mass index <20 and any degree of weight loss >2%; or ‐ Appendicular skeletal muscle index consistent with sarcopenia (male patients <7,26kg/m^2^, female patients <5,45kg/m^2^) and any degree of weight loss >2% (According to international consensus by Fearon *et al*., 2011)^2^
Sarcopenia	Syndrome characterized by progressive and generalized loss of skeletal muscle mass and strength with a risk of adverse outcomes such as physical disability, poor quality of life and death^148^, ^182^	‐ Reduction of anabolic hormones ‐ Increased apoptotic activities in the muscle ‐ Systemic low‐grade inflammation ‐ Mitochondrial dysfunction ‐ Impaired regeneration of muscle cells 35, 153, 154, 155, 163, 171, 175	‐ Criterion 1: Low muscle strength (assessed by grip strength; chair stand test); ‐ Criterion 2: Low muscle quantity or quality (ASMM by DXA; whole‐body SMM or ASMM by BIA; lumbar muscle cross‐sectional area by CT/MRI); ‐ Criterion 3: Low physical performance (Gait speed; SPPB; TUG; 400‐meter walk). (Probable sarcopenia is identified by Criterion 1. Diagnosis is confirmed by additional documentation of Criterion 2. If all the three criteria met, sarcopenia is considered severe.) (According to EWGSOP2)^148^, ^182^
Anorexia	Loss of apetite^195^	‐ Loss of weight and fat mass, that can be reversed by nutritional support ‐ Higher and/or persistent inflammatory response ‐ Disruption of neuronal pathways that regulates eating behaviour 199, 201, 204	Based in visual analogue scales, numerical scales, verbal descriptors or individual questionnaires (EORTC).^17^, ^207^ Two methods have been suggested: FAACT‐A/CS and VAS.^207^, ^220^
Asthenia	Condition defined as absence of strength, weakness and reduced vital power^224^	‐ Generalized weakness and fatigue ‐ Loss of muscle force, muscle weakness ‐ Profound tiredness after usual or small effort ‐ Decreased in intellectual work ‐ Impaired concentration and loss of memory ‐ Emotional lability ‐ Inflammation (cytokines) ‐ In cancer patients: combination of factors released by the tumour and direct consequences of the tumour presence 17, 223, 225, 226	Assessment hard, difficult and subjective. ‐ Assess the functional capacity through the capacity of do standard tasks. ‐ Assessment of performance status by scales to rate the functional abilities. The most two used scales are ECOG‐PS and KPS. ‐ Subjective assessment of fatigue through questionnaires 17, 234, 235, 236, 237, 238

Several diagnostic criteria have been developed for these conditions. While the diagnosis of sarcopenia relies on the evaluation of muscle mass and function, diagnosing cachexia requires an assessment of skeletal muscle and total body mass. While cachexia and sarcopenia have been well studied and some degree of consensus has been reached concerning both, secondary anorexia and asthenia have been largely forgotten or ignored.

The terms used for designating and characterizing these conditions have evolved over the years (e.g. asthenia and fatigue), which may cause some degree of confusion. In fact, in a general way, the terms to designate the conditions must be simple and unanimous.

The importance of better understanding the differences between these conditions resides in the fact that it can allow clinicians to accurately diagnose the patients. The molecular pathways that are involved in these conditions and syndromes also must be better explored in order to improve diagnosis and identify therapeutic targets. An early and accurate diagnosis can make the difference in the treatment options as well in the patients' outcome and quality of life.

## Conflict of interest

None declared.

The manuscript does not contain clinical studies or patient data.
